# Enhanced Optical Efficiency and Carrier Dynamics in InGaN/GaN Light-Emitting-Diode Structures through Combination of Superlattice- and Prewell-Strain Control Strategy

**DOI:** 10.1021/acsami.6c06408

**Published:** 2026-06-25

**Authors:** Fatimah Alreshidi, Lih-Ren Chen, Hadeel Alamoudi, Dhaifallah R. Almalawi, Nimer Wehbe, Georgian Melinte, Tien-Chang Lu, Iman S. Roqan

**Affiliations:** † Physical Sciences and Engineering Division, 127355 King Abdullah University of Science and Technology (KAUST), Thuwal 23955-6900, Saudi Arabia; ‡ Department of Photonics, College of Electrical and Computer Engineering, 598129 National Yang Ming Chiao Tung University, Hsinchu 30010, Taiwan; § Department of Physics, College of Science, Taif University, Taif 21944, Saudi Arabia; ∥ Imaging and Characterization Core Laboratory, King Abdullah University of Science and Technology, Thuwal 23955-6900, Saudi Arabia

**Keywords:** structure, superlattices, III-nitrides, light emitting devices, carrier dynamic, optical characterizations

## Abstract

We explore the effect of combining two distinct strain-engineering multilayer regions (designated as L1 and L2) on the structural quality, carrier dynamics, and device optical efficiency of InGaN/GaN multiple quantum well (MQW)-based light-emitting diode (LED) structures. In S1, L2 consists of a single ultrathin InGaN/GaN layer, whereas in the S2 LED structure, L2 contains several superlattice (SL) pairs beneath the MQWs. The incorporation of superlattices in L2 leads to a significant enhancement in the optical performance in the S2 LED structure relative to S1. A detailed structural analysis indicates that the dimensions of V-pits, including their size, depth, and sidewall thickness, are determined by the configuration of the L2 region. The presence of SLs in S2 promotes a more homogeneous indium distribution within the InGaN quantum wells, whereas S1 exhibits In-rich nanosegregation. In S2, a monotonic decrease in indium content from the bottom to the top of the MQWs adjacent to the V-pits is observed, attributed to the fully pseudomorphic strain regime. Photoluminescence (PL) and time-resolved PL measurements show that S2 achieves an internal quantum efficiency exceeding 85% and exhibits distinct drooping characteristics under high carrier injection. These results indicate that carrier overflow and band-filling effects facilitate the transfer of carriers to higher energy states associated with the V-pit sidewalls, followed by repopulation of the main MQWs occurring through regions with gradually varying indium content, resulting in dominant radiative recombination in S2 at room temperature. Electroluminescence measurements confirm that the S2 LED attains an external quantum efficiency 1.6 times greater and an output power approximately 66% higher than those of S1.

## Graphical abstract

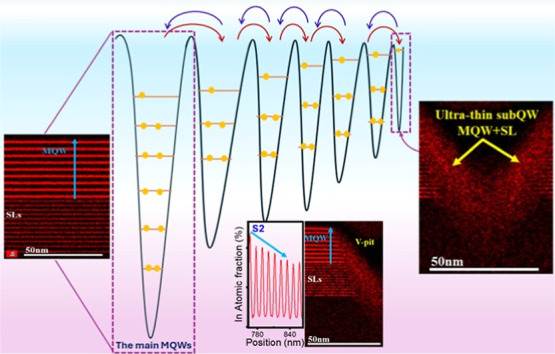

## 1 Introduction

High-efficiency emitting devices based on III-nitride multiple quantum wells (MQWs) exhibit excellent properties, such as a direct and wide bandgap, as well as optical tuneability, leading to a wide-range optoelectronic applications, mainly for energy-efficient lighting applicaitons.^1–3^ However, InGaN MQW-based LEDs in particular still suffer from several challenges that hinder efficient radiative recombination and thus lower the internal quantum efficiency (IQE).^4,5^ These issues arise because of the large lattice mismatch between InN and GaN which lead to substantial strain within the QWs, generating a high density of threading dislocations (TDs) that penetrate the MQW active layer and act as nonradiative recombination centers, resulting IQE reduction.^6–8^

Several approaches have been adopted to mitigate these issues. For example, the light-emitting diode (LED) structures have been optimized^9–12^ by strain engineering to suppress the nonradiative recombination associated with TDs, thereby significantly enhancing the efficiency of InGaN/GaN MQW-based optoelectronic devices. These approaches, however, require the introduction of prestrain engineering InGaN layers such as superlattice (SL) layers^13,14^ beneath the MQW with the aim of reducing the number of TDs penetrating the MQW region while enhancing homogeneous In incorporation within the quantum well (QW) layers.^15,16^ Such layers allow a formation of V-pits^17–19^ initiating below the MQWs to reduce further TD penetration into the active layer. Indium segregation, or InN nonuniform compositional fluctuation in the InGaN QW layer, increases the separation of electron and hole wave functions in MQWs, resulting in a significant reduction in the radiative recombination rate and the IQE values.^20^

According to the extant studies, the size of such V-pits plays a significant role in enhancing the InGaN-based LED performance. When the V-pit size increases, the nonradiative recombination process is further suppressed,^21,22^ while the hole injection from *p*-GaN into the QWs increases.^21,23^ Furthermore, the V-pit sidewalls (characterized by much thinner QWs with lower In content than the main MQWs) formed on the {10-11} facet play a crucial role in improving the radiative recombination rate, as they act as energy barriers due to their lower In content, thus preventing carriers from recombining nonradiatively.^9,15,21^ Therefore, V-pit size and wall thickness must be controlled by, for example, introducing further ultrathin InGaN pre-multilayers underneath the SLs, which can enhance strain engineering. However, the effect of a combination of two strain-engineering regions comprising multiple ultrathin layers with different structures on the device optical efficiency and carrier dynamics is still insufficiently studied, even though its understanding is crucial for optimizing the efficiency of InGaN-based LEDs.

In this work, we are addressing this gap in extant literature by demonstrating the effect of two strain-engineering layers—(1) a premultilayer set (L1) consisting of multiple thin InGaN layers located below (2) the second region (L2) that comprises multiple InGaN/GaN SLs—on the structural and optical properties of InGaN/GaN MQWs-based LED structures. For comparison, we study a second sample that possesses a similar L1 structure, but its L2 structure comprises a single ultrathin InGaN/GaN (SL) layer to elucidate the effects of different strain-engineering strategies.

## 2 Experimental Methods

### 2.1 Sample Growth

Two LED structures were grown on *c*-plane patterned sapphire substrates (PSS) by metal–organic chemical vapor deposition (MOCVD). The growth process commenced with the deposition of an AlN buffer layer approximately 10 nm thick on top of the PSS. Two sets of strain-engineering regions were incorporated beneath the In_0.14_Ga_0.86_N/GaN MQWs. The first set (L1) consists of five pairs of InGaN/GaN pre-multilayers. This is followed by a second set (L2), which comprises a single SL for the S1 LED structure and several SLs for S2.

The active region comprises nine InGaN/GaN MQW pairs for both samples, as shown in the schematic diagram of their LED structure, shown in Figure 1a, and its corresponding STEM image presented in Figure 1b. The growth details have been published elsewhere.^15^

**Figure 1:**
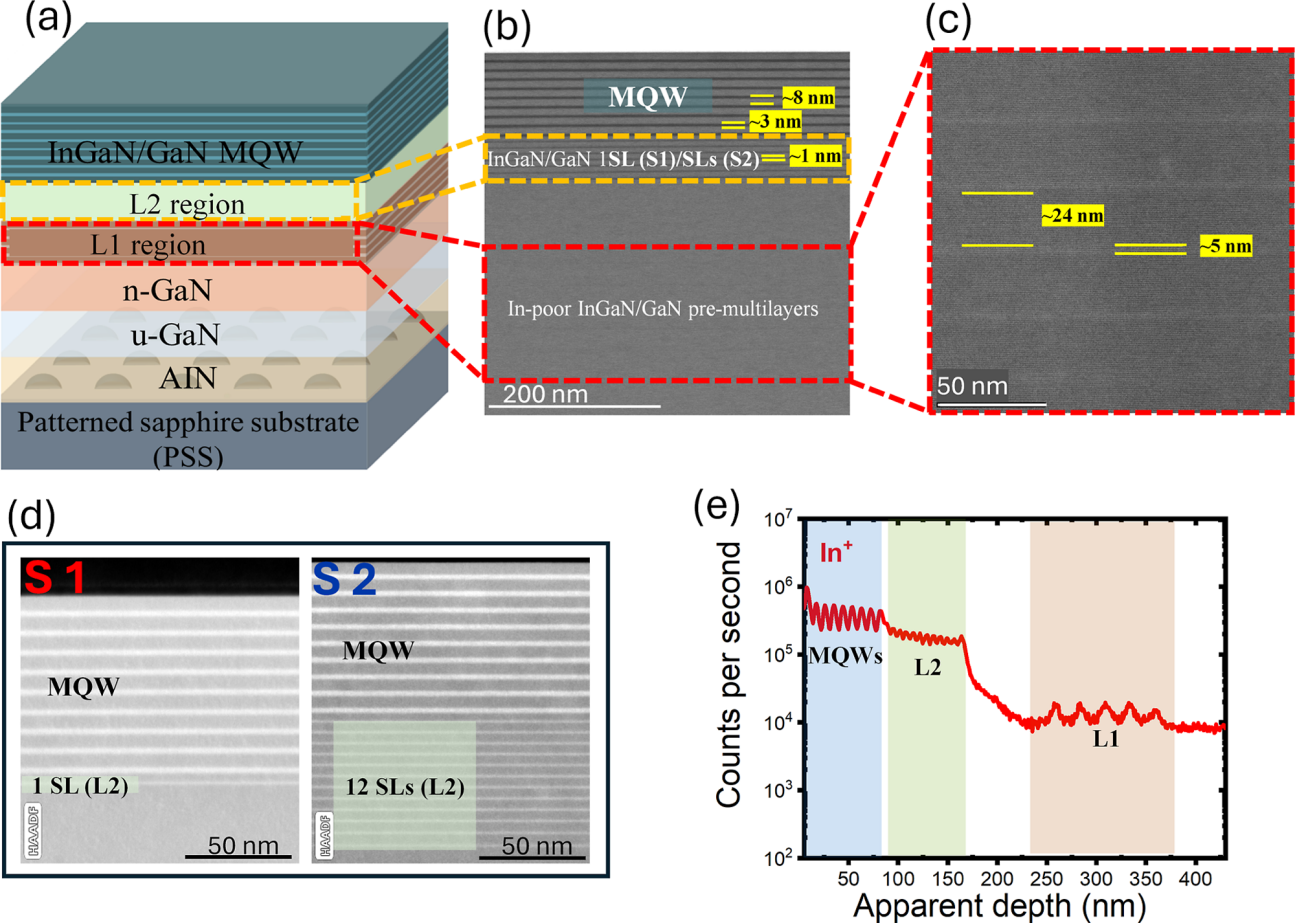
(a) Schematic diagram of the InGaN/GaN MQW LED structure. STEM cross-sectional images of (b) MQWs and the two sets of the strain-engineering layers; (c) first pre-multilayers, L1, set (surrounded by red in (b)); (d) second strain-engineering layer, L2, set comprises a single SL layer in S1 (left panel) and 12 SL pairs (right panel) in S2; and (e) secondary ion mass spectrometry (SIMS) profile of In contents in L1, L2, and MQW regions for S2.

### 2.2 Device Fabrication and Characterizations

The LED epitaxial structures were grown with a nucleation layer, followed by an undoped GaN template, an n-type GaN layer, prestrained layers, an MQW active region, and a *p*-type GaN cladding layer. Then, as-grown wafers underwent chip processing. The p-contact consisted of an ITO transparent layer together with a finger-shaped metal alloy, and both the p-type and n-type metal contacts consisted of Cr/Al/Cr/Au layers deposited by electron-beam evaporation. The processed wafers were diced into chips with dimensions of 250 × 580 μm^2^. Device characterization was carried out under continuous-wave current injection at room temperature (RT), and electroluminescence (EL) was measured using an integrating sphere.

### 2.3 Structural Characterizations

High-Resolution Scanning Transmission Electron Microscopy (HR-STEM) was performed on a Titian TEM, Energy Dispersive X-ray Spectroscopy (EDX). SIMS. RT Raman spectra were measured to study the strain effect differences between the samples using the Witec Apyron confocal Raman system equipped with an excitation source of 532 nm with a 50× objective.

### 2.4 Optical Characterizations

We employed RT power-dependent (PDPL) (the injection carrier rate ranging from 1.89 × 10^32^ to 1.63 × 10^33^ cm^–2^ s^–1^) and temperature-dependent (TDPL) photoluminescence (5 to 300 K) to investigate optical efficiency and IQE. Time-resolved photoluminescence (TRPL) measurements captured by the Hamamatsu streak camera were utilized under the streak camera’s single sweep mode using a pulse picker to control the pulse configuration according to the sample emission decay to study the carrier dynamics at different powers and temperatures (for evaluating radiative and nonradiative recombination lifetimes). The samples (located in a Janis He closed cryostat) were excited by the second harmonic (λ = 400 nm) of a mode-locked Coherent Ti/sapphire femtosecond ultrafast laser output at 800 nm using a second harmonic generator.

## 3 Results and Discussion

Figure 1b,c shows the STEM cross-sectional images of the first preset (L1) that consists of five InGaN/GaN pairs for both LED structures, while the second configuration (L2) differs, whereby a single In-poor InGaN/GaN SL is adopted for the S1 structure and 12 SLs for the S2 structure, as shown in Figure 1d. The structural composition and In distribution of the two samples (S1 and S2) were evaluated via SIMS depth profiling. The structure dimensions and In distributions of L1 and L2 compared to those of MQWs were confirmed by SIMS analyses of In content, as shown in Figure 1e. Figure S1 in Supporting Information shows SIMS elemental profiles of (In, Ga, and N) as a function of depth for both LED structures, indicating further the difference in the two LED structures. As illustrated in relation to S1, the In signal reveals three distinct growth regimes in the S1 structure. The primary MQW region (from the surface to the depth of ∼85 nm) exhibits the highest In concentration (*x* = ∼0.14), characterized by peak intensities approaching 10^6^ counts/s. The intermediate layer (L2 = SLs for S2) contains moderate In levels, which is more than 50% lower than in the MQW, as shown in Figure 1e. Finally, five pre-multilayer (L1) are placed at 160–234 nm (S1) and 250–365 nm (S2) depths, as indicated by SIMS profiles of both structures in Figure S1. While the L1 region has the lowest In content (approximately 2 orders of magnitude lower than the MQW, it features the largest interlayer (∼24 nm GaN) spacing between the 5 nm InGaN multilayers. However, the In content of L1 in S1 structure is slightly higher than that in S2.

### 3.1 Analyses of the Strain

To elucidate crystalline quality, both samples were subjected to confocal micro-Raman measurements to analyze the strain effect. As shown in Figure 2 (and Figure S2 illustrating the peak fitting), two prominent wurtzite GaN peaks—the E_2_ (high) mode and a peak related to the InGaN E_2_ (high) mode^24,25^ are observed in the spectra obtained for both samples. The GaN E_2_(high) related peak is attributed to strain on the layer, which shifts to a higher value (blueshift) when the strain is compressive or a lower value (redshift) when the strain is tensile compared to that in unstrained bulk film (567 cm^–1^).^26–29^ Raman spectra illustrated in Figure 2a,b reveal that the GaN layer underneath the L1 and L2 regions is under compressive strain^29,30^ for both samples, as the E2 (high) peak has shifted to a higher value (∼569.76 cm^–1^) for S1 and even higher (∼570.107 cm^–1^) for S2.

**Figure 2:**
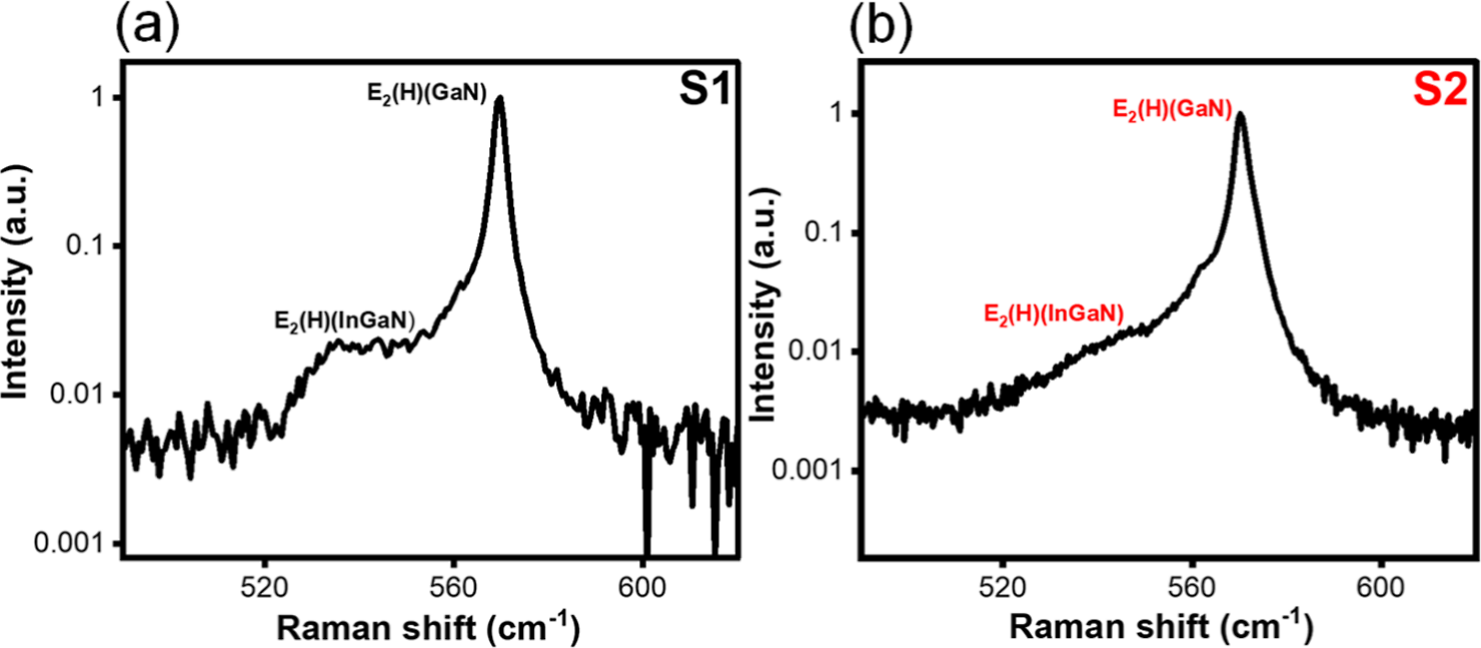
RT μ-Raman spectra of (a) S1 and (b) S2.

Based on the reciprocal space mapping (RSM) results (see Figure S3 in Supporting Information), the dominant intensity peak for all samples originates from the GaN layer, with four MQW satellite peaks (0th to third order) appearing at lower diffraction angles. In S2, the precise vertical alignment of the GaN and MQW satellite peaks along the perpendicular line indicates that the in-plane lattice constants are matched (i.e., *a*_InGaN_ = *a*_GaN_), maintaining coherence with the GaN template, which confirms that S2 is fully strained pseudomorphic.^31^ However, S1 exhibits the weaker satellite intensity, indicating less or partially pseudomorphic strain, as evidenced by a distinct lateral shift of the MQW peaks away from the GaN reciprocal lattice point, indicating a very slight partial relaxation in S1.^32–34^ These findings are supported by the Raman results as well as STEM analyses, as will be shown later.

### 3.2 HR-STEM Structural Analyses

To further study the effect of incorporating L1 and L2 on the In fluctuation within the MQWs, HR-STEM images and corresponding EDX profiles were obtained for both LED structures. Figure 3a illustrates the In EDX maps (top panels) and the corresponding profiles (bottom panels) for both structures, which clearly show MQWs and L2 layers. The corresponding In profile (taken from the marked area with yellow arrows in the top panels of Figure 3a) indicates the same In contents in all MQW layers for both samples, while the In content remains uniform in the SLs comprising S2.

**Figure 3:**
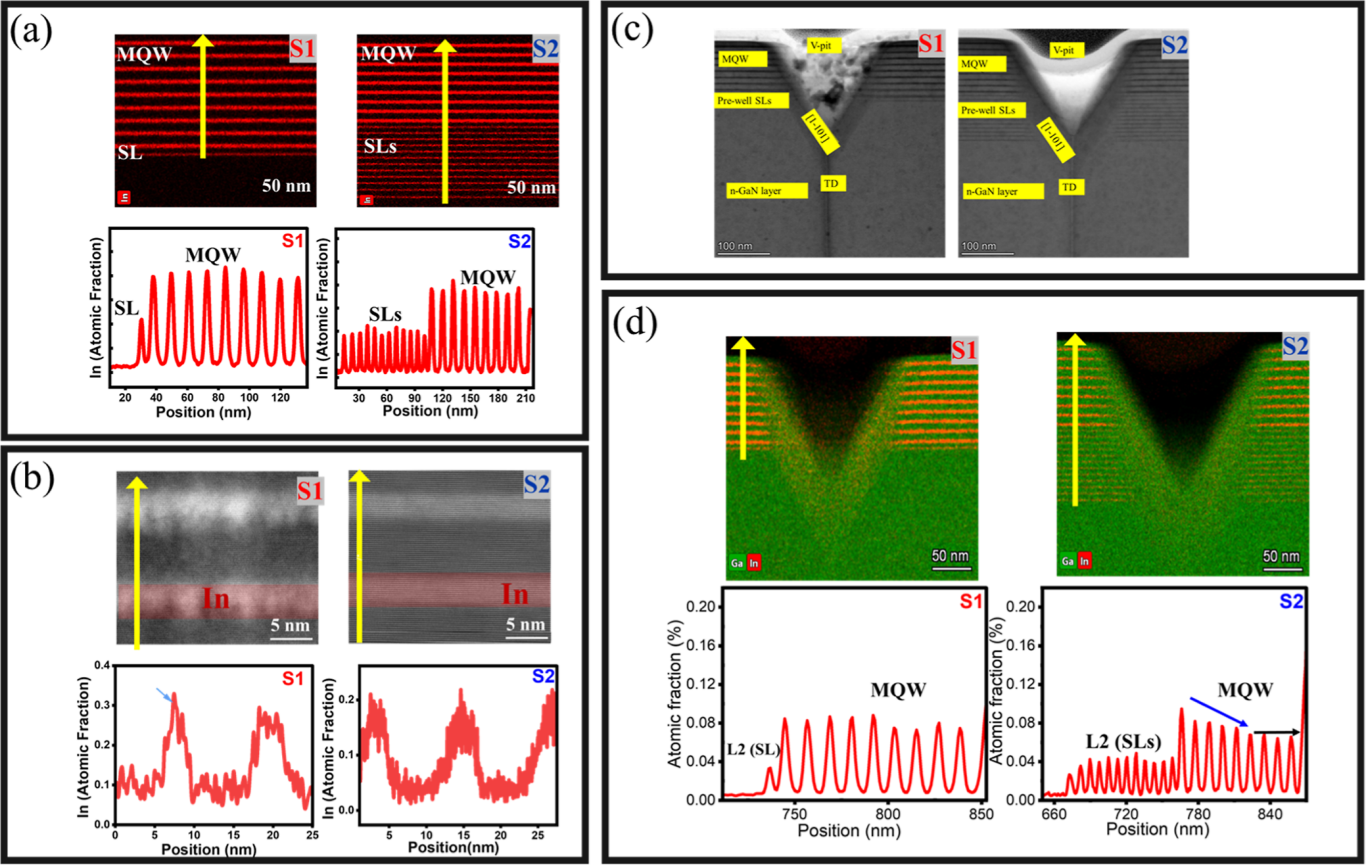
(a) EDX (top) and the In content profiles (bottom) taken from regions away from V-pits on the active region (MQW) + L2 (SLs pairs) and (b) HR-STEM (top) and In EDX profile (bottom) for S1 and S2 on the active region (MQW); (c) Cross-sectional STEM images for S1 and S2; and (d) EDX maps (top) and corresponding In and Ga compositional profiles (bottom) across the MQW and SLs layers, for S1 (left panels) and S2 (right panels). (The yellow arrows in (a,b,d) indicate the area where the corresponding EDX profiles were measured).

The nanoscale segregation in the HR-STEM image illustrated in the top-left panel of Figure 3b and its corresponding In profile (bottom-left panel of Figure 3b) show obvious InN fluctuation within the QWs in the S1 structure (single SL in L2). This finding is confirmed by the presence of higher strain that can drive In atoms to cluster together to relieve local strain,^35^ forming In-rich quantum dot (QD)-like regions within the QW layers. In-rich nanosegregation can result in rougher interfaces and local In fluctuations in well, as confirmed by the EDX profile (blue arrow of EDX profile in the left-bottom panel of Figure 3b), which can impact the optical behavior of the LED. On the other hand, the use of 12 SL pairs in L2 alongside L1 pre-multilayers results in a more homogeneous In distribution within the QW, no In fluctuation is observed, as confirmed by the HR-STEM image of S2MQWs shown in Figure 3b (top-right panel) and the corresponding profile (bottom-right panel). This evidence confirms that strain engineering resulting from the introduction of SL layers into the structure plays a significant role in optimizing the strain and thus enabling more homogeneous In distribution within InGaN MQWs.

STEM images illustrated in Figure 3c show the V-pit is much deeper and larger in S2 (right panel) compared to S1 (left panel). Controlling the In content and InGaN thickness in L1 and L2 sets allows the V-pits to be initiated below the L2 region in both samples. As can be seen from cross-sectional STEM images presented in Figure 3c, V-pits tend to be initiated at the top of TD sites on the GaN layer above L1. However, as the size of these V-pits is significantly influenced by the number of SL pairs, it is greater in S2 (∼275 nm) compared to S1 (∼212 nm). However, given that S1 contains a single In-poor InGaN SL in the L2 set, the 212 nm V-pit^15^ size is still considered large compared to that in the literature and was achieved by pre-straining L1, which forces V-pits to be opened before reaching the SLs. In addition, the V-pit sidewalls comprise both ultrathin MQWs and SLs (with a <1 nm QW thickness), as shown in STEM images as well as the corresponding map in Figure 3d. Such behavior is due to the growth in the {10-11} facet of the V-pit sidewalls, resulting in different growth rates.^36^

To further analyze the In distribution across the MQW + SLs structure near the V-pits, we captured EDX profiles taken from the area pointed by the yellow arrows in EDX maps (top panels in Figure 3d) for both structures. As shown in the bottom-right panel of Figure 3d, the EDX profile indicates that, for S2, the In concentration decreases gradually from the first bottommost MQW layer to the fifth (as illustrated by a blue arrow). This gradual decrease in the In contents (as moving from the bottom to the fifth layer above, as shown by the yellow arrow in the EDX map of S2 illustrated in the top panels of Figure 3d) near the V-pits could be attributed to a more homogeneous strain engineering mechanism accompanied by V-pit formation compared to that in S1. As the V-pit completely opens at the top, the strain in the MQW is slightly different^37^ compared to that when it is partially opened. On the other hand, when the V-pits start opening (which is in our case, below SLs), the MQWs strain remains (fully pseudomorphic), resulting in further In incorporation in the bottommost QW layer. It then decreases in the five MQWs above, finally reaching saturation in the subsequent QW layers where the In content remains constant (black arrows) when the V-pit is not fully opened. When the strain is lower, the driving force for In incorporation leads to a lower In content incorporated into InGaN QWs. However, such behavior is absent for MQWs in S1, as it is partially pseudomorphic (see the In profile presented in the bottom-left panel of Figure 3d taken at the similar area near V-pits).

### 3.3 Power-Dependent Carrier Dynamic Analyses

PDPL measurements were conducted to determine the optical behavior and the IQE as a result of incorporating different L2 configurations (i.e., 12 SLs compared to a single SL). Figure 4a shows changes in the RT PDPL spectra produced for S1 and S2 at a 400 nm excitation wavelength (above the InGaN bandgap but below the GaN QB) as the power density increases. A 2-fold enhancement in the emission at ∼467 nm is observed for S2 compared to S1. Using the ABC model, the IQE was calculated via PDPL measurements, and the total carrier injection rate (*G*) was derived as follows^38^

1 $${G=An+Bn}^{2}+{Cn}^{3}$$

where *A*, *B*, and *C* are the corresponding Shockley-Read-Hall (SRH) nonradiative, radiative, and Auger recombination coefficients, respectively,^39,40^ the IQE calculation methodology has been published elsewhere^41^ as in eq 2

2 $$IQE=\frac{{Bn}^{2}}{G}$$

In Figure 4b, IQE reaches its maximum (∼85%) at *G* = 3.6 × 10^32^ cm^–3^ s^–1^ for S2, whereas IQE maximum (∼62%) is obtained for S1 at 1 order of magnitude higher *G* = 1.65 × 10^33^ cm^–3^ s^–1^.

**Figure 4:**
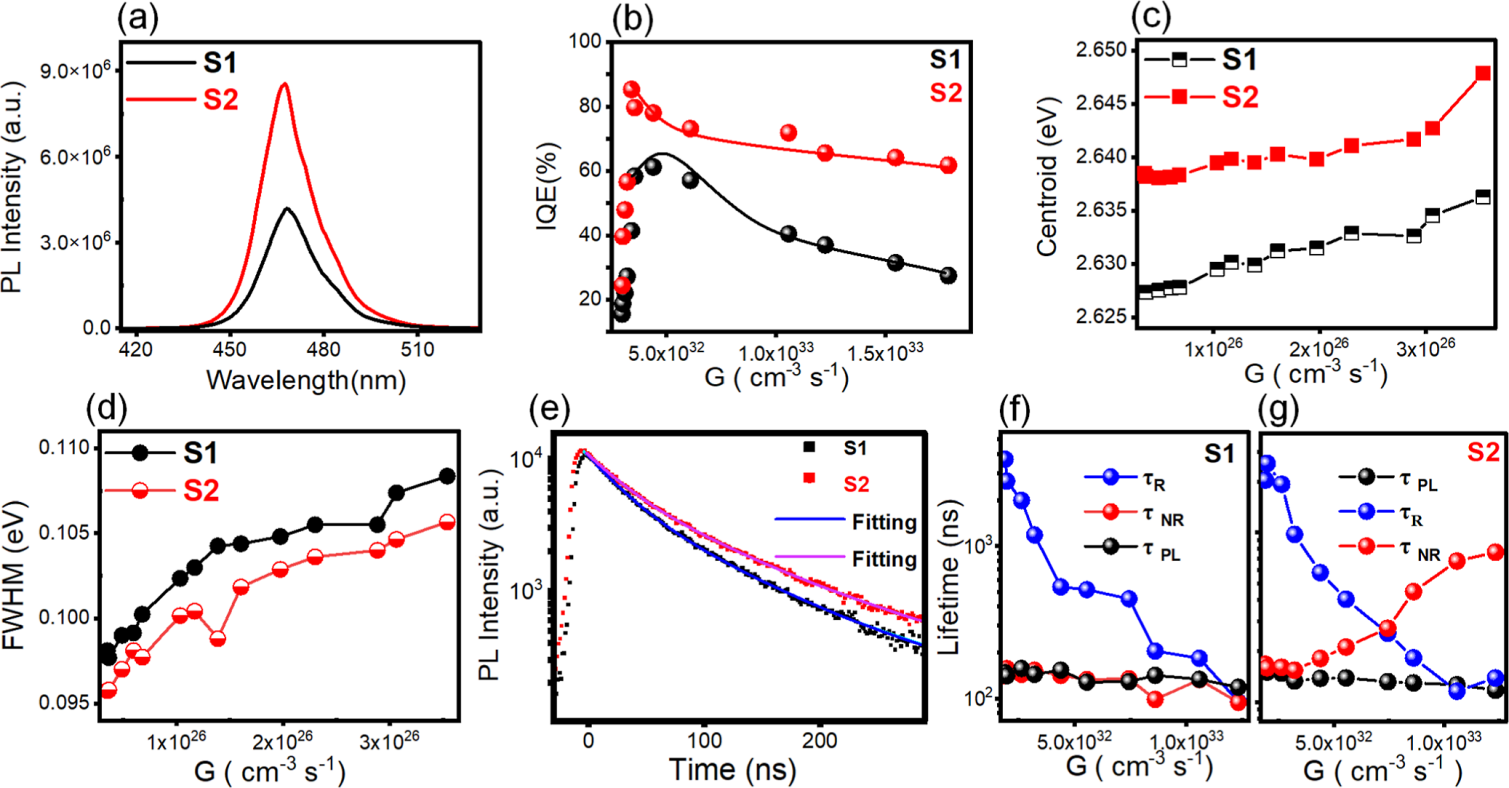
(a) PL spectra captured at RT for S1 and S2; (b) IQE; (c) peak positions; (d) fwhm as functions of G, for both structures; (e) RT TRPL excited for G = 1.23 × 10^33^ cm^–3^ s^–1^; and radiative (τ_R_) and nonradiative (τ_NR_) recombination lifetimes compared to the total lifetime of slow decay (τ_PL_) as functions of *G* for (f) S1 and (g) S2.

Figure 4b further shows that the efficiency droop characteristics of both samples behave differently as *G* increases. According to Seong et al.,^42^ the IQE behavior as a function of injected carrier density can be determined from its shape (concave, convex, or linear), as each shape is associated with a different dominant recombination mechanism. Specifically, the Type 1 (concave) IQE curve obtained when carrier injection increases, which exhibits a steep decline due to strong carrier overflow or band filling from the QWs. This behavior results in a rapid efficiency droop as excess carriers escape confinement and recombine nonradiatively. On the other hand, if a Type 2 (convex) IQE curve is obtained when *G* increases rapidly and then saturates, this is due to SRH nonradiative recombination via defects, after which IQE declines slowly, while Type 3 (linear) is due to electron tunneling from inside MQWs to the top layer (e.g., *p*-type layer).^42^

The IQE droop observed in S1 exhibits a convex dependence for low G values (<1 × 10^33^ cm^–3^ s^–1^), which can be attributed to dominant nonradiative recombination at defect sites via the SRH process.^42^ This behavior might be associated with In-rich nanosegregation, as observed by STEM images, or nanoclusters, within the quantum wells, as revealed by HR-STEM analysis. These nanoclusters serve as barriers between carriers and defects. As G increases (>1 × 10^33^ cm^–3^ s^–1^), carriers acquire sufficient energy to escape from the nanoclusters, leading to a linear decrease in IQE that reaches 27.5% for S1. The observed linearity at higher G values suggests that escaped electrons tunnel from within the quantum well to shallower states, such as those formed by V-pit-induced subquantum wells.

On the other hand, the IQE of S2 follows a concave IQE curve whereby, as *G* increases, more and more electrons are injected into the QWs. Still, even at high *G* (>0.6 × 10^32^ cm^–3^ s^–1^), the electron-confining potential barrier is not high enough to prevent electrons from overflowing out of the main QW states into In-poor sub-QWs formed by ultrathin QWs (∼1 nm) of the V-pit sidewalls, which are characterized by higher energy (shallower) states than the main MQWs, then nonradiative recombination increases at such high G. In addition, gradual In content decrease within the top five MQWs near the V-pit (shown in the EDX profile of Figure 4d) can assist in such a process, resulting in a ∼61.8% IQE for S2.

Such a superior IQE value of LED S2 is due to the combined strain engineering effect of the optimized number of layers in L2 as well as prestrained layers in L1.^12,19^ In an earlier study focusing on an LED structure based on InGaN/GaN with different numbers of SLs (10, 15, 30, and 60 pairs), the sample with 15 SL pairs was found to have the most optimal IQE (70%)^12^ Okada et al. similarly investigated the impact of varying SL periods—specifically 5, 10, and 20 pairs—on the IQE. Their results demonstrated that the 10 SL pairs yielded an IQE of approximately 55%.^43^ However, Liu et al. reported that a larger SL number (24 pairs) was used in a green LED structure to obtain an optimum IQE value of ∼29%.^19^

To gain further insight into the origin of the observed IQE behavior, peak positions and full width at half-maximum (fwhm) values were analyzed as a function of *G*. As indicated in Figure 4c, the peak located at ∼2.638 (∼470 nm) emitted by S2 is blueshifted by ∼12 meV compared to that of S1 (2.627 eV) as S2 is fully strained.^44^ Furthermore, as *G* increases, the PL peak exhibits a blueshift for both samples, but the shift is greater (10 meV) in S1 characterized by a single SL in L2 than in S2 containing L2 with 12 SLs. This larger shift with an increase in *G* can be due to In-rich InGaN segregation in S1, as indicated in the STEM image presented in Figure 3b, showing the presence of In-rich nanosegregations inside the MQWs of S1, which act as QD-like centers that might provide deeper localization states. For S2, a slight peak blueshift (∼4 meV) occurs as *G* increases up to 3 × 10^26^ cm^–3^ s^–1^, but once *G* exceeds 3.1 × 10^26^ cm^–3^ s^–1^, further blueshift (∼5 meV) occurs rapidly.

The peak fwhm behavior further elucidates our analyses. For both structures, the peak blueshift accompanied by peak broadening as *G* increases was attributed to the band-filling or overflow effect^45^ in high-energy localized centers under high carrier injection and a negligible QCSE.^45^ A clear dip in fwhm is observed for S1 when *G* reaches 1.38 × 10^26^ cm^–3^ s^–1^, which can be due to strong carrier localization in In-rich segregation within the MQWs. Thus, as carriers are released with a further increase in *G*, fwhm exhibits V-shape behavior as *G* increases. Such behavior was not observed in S2, which was confirmed to have homogeneous InGaN content inside the QW.

To investigate the effect of a combination of these two strain-engineering regions on the carrier dynamics, TRPL measurements were carried under 400 nm excitation. Figure 4e shows a different carrier lifetime decay at RT in S1 compared to S2, indicating that the carrier relaxation process follows different pathways for each structure. We found the best-fitting curve for the PL lifetime decays showing biexponential behavior. The biexponential decay implies multistate recombination, suggesting that the excess carrier recombination occurs via multiple pathways^46^ and can be mathematically described by the following expression^45,47^

3 $$I\left(t\right)=A1\exp\left(-\frac{t}{\tau_{1}}\right)+A2\exp\left(-\frac{t}{\tau 2}\right)$$

where *A*1 and *A*2 are the fast- and slow-decay intensity peak at time *t* = 0, respectively, while τ_1_ and τ_2_ are the fast and slow decay times, respectively. As shown in Figure 4f,g (the slow component) and Figure S4 (the fast componenet) for S1 and S2, respectively, each of these components can be resolved radiatively and nonradiatively using^46^

4 $$\tau_{Rad}=\frac{\tau_{total}\left(T\right)}{IQE},{,\tau }_{non‐rad}=\frac{\tau_{total}\left(T\right)}{1-IQE}$$

where τ_slow_ is given in eq 3. The total PL lifetime (τ_PL_) as a function of *G* at RT, along with the radiative (τ_Rad_) and nonradiative (τ_nonrad_) lifetimes of the slow component as *G* increases, for both samples. (As shown in Figure S4, the fast component exhibits similar behavior of radiative and nonradiative contributions). For S1, the radiative lifetime decreases continuously, whereas the nonradiative lifetime remains constant as *G* increases, confirming that radiative recombination is dominant at low excitation power. However, as the carrier injection increases, there is competition between radiative and nonradiative recombination, confirming that the observed S1 IQE droop is due to the SRH process. On the other hand, for S2, the radiative recombination lifetime decreases as *G* increases, while the nonradiative component increases, indicating that the former recombination mechanism becomes more dominant at high carrier injection values. This observation supports our hypothesis that the observed droop behavior can be attributed to overflow accompanied by the band-filling process.^42^

### 3.4 Temperature-Dependent Carrier Dynamics Analyses

Figure 5a,b shows the 5 K and RT PL spectra of S1 and S2, respectively. Both samples exhibit a blueshift in their main PL peak energy as the temperature increases from 5 to 300 K. The magnitude of the blueshift is greater for S2 (0.04 eV) compared to S1 (0.02 eV), which could be due to the band-filling and overflow effect, as suggested by PDPL and PD-TRPL findings. An additional peak appears at ∼420 nm in the S2 spectrum, which is due to the ultrathin layers of MQWs + SLs comprising V-pit sidewalls (as also shown in Figure S5 in Supporting Information for PL spectra), because of their narrower QW width and higher quantized levels as well as their wider bandgap due to lower In contents.^15^ At 5 K both samples exhibit a phonon replica at the low-energy side of the spectra end (>460 nm). We calculated the ratio of integrated PL intensity at 300 K to that at 5 K from the TDPL measurements to be 60% and 87% for S1 and S2, respectively, which agrees well with the IQE findings obtained using the ABC method, confirming the superior efficiency of S2 as an LED structure.

**Figure 5:**
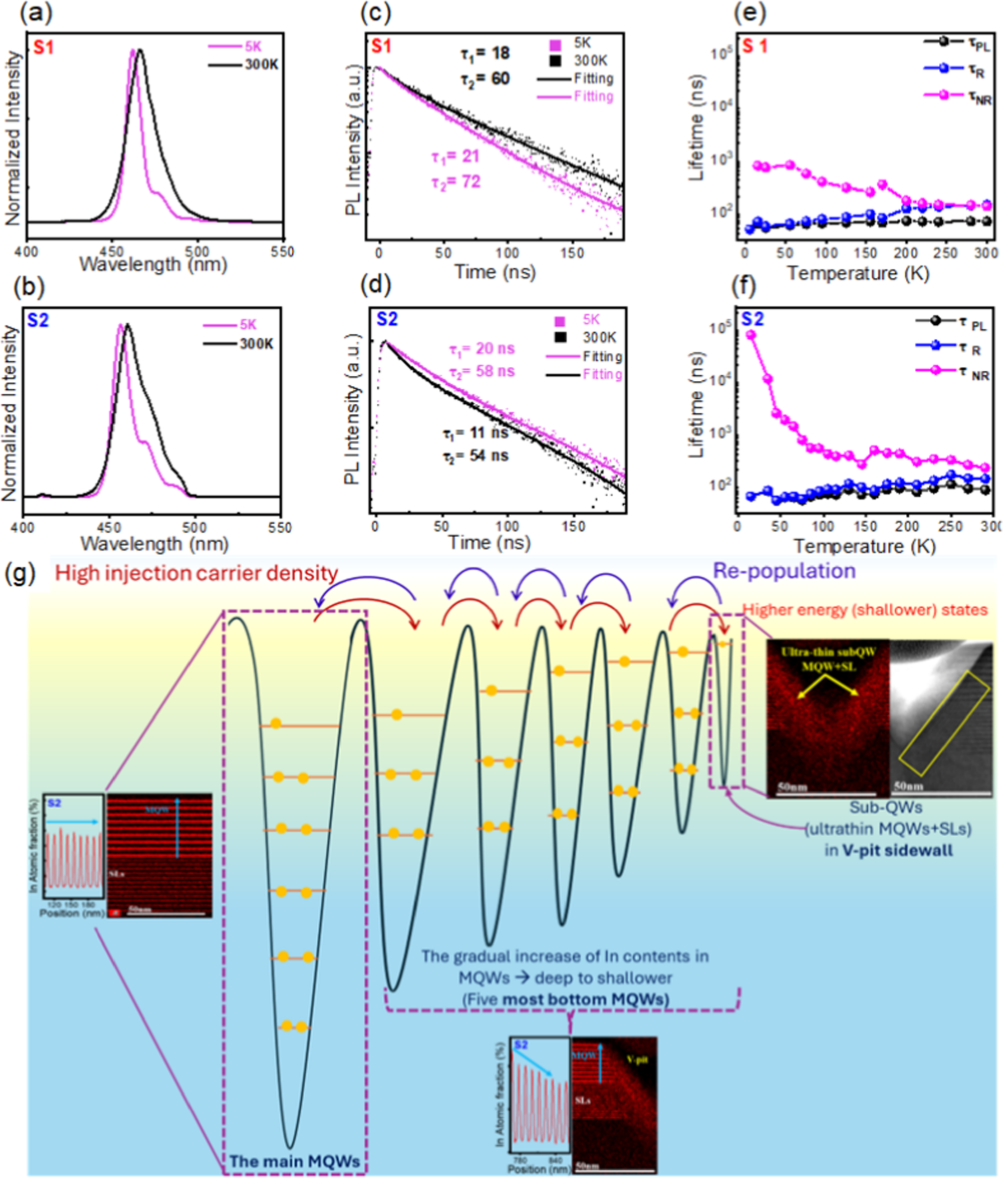
PL spectra of the MQWs peak at 5 K and RT for (a) S1 and (b) S2 under maximum injection power density; and TRPL at 5 K and RT for (c) S1 and (d) S2 excited by 400 nm**.** Lifetime decay τ_R_ and τ_NR_ compared to the total τ_PL_ of slow component vs temperature for (e) S1 and (f) S2. (g) Schematic diagram of the overflow and band filling mechanism between the MQWs and sub-QWs of V-pit sidewall assisted by MQWs characterized by a gradual decrease of In content near V pits.

The TRPL spectra of InGaN/GaN MQWs captured for both samples at RT and 5 K and fitted using the biexponential function given in eq 2 are shown in Figure 5c,d. As the nonradiative centers are activated by increasing temperature, a considerable change in the total lifetime values (Δτ = 12 ns) can be noted for S1, showing more substantial nonradiative recombination contributions at RT compared to those at 5 K. Surprisingly, for S2, the lifetime measured at RT exhibits a slight change (Δτ = 4 ns) compared to that measured at 5 K, suggesting a minor contribution of nonradiative recombination as temperature increases. These findings provide further evidence of the dominant contribution of the radiative recombination process at RT, concurring with the PD-TRPL results and demonstrating superior S2 efficiency.

As indicated by the TRPL results, S2 exhibits faster carrier lifetimes (Figure 5d) at RT than those of S1 (Figure 5c). In other words, S2 is characterized by a higher recombination rate relative to S1, signifying enhancement of the radiative recombination process through a more effective carrier injection into the QWs and a reduction of defect density.

Figure 5e,f shows the TD-TRPL measurements at high carrier injection density (maximum *G*), further demonstrating that the radiative recombination rate in S2 is dominant across the entire temperature range from 6 to 300 K (i.e., the radiative recombination remains the primary process). In contrast, S1 shows a crossover at 250 K, indicating that nonradiative recombination becomes dominant at 300 K. Interestingly, for S2, at a low temperature range <100 K, the nonradiative lifetime (τ_NR_) is significantly longer—over 1000 times longer at 5 K—compared to the total lifetime, as shown in Figure 5f. This indicates that nonradiative recombination is negligible at low temperatures. Above 150 K, however, τ_R_ remains dominant. Conversely, for S1, the τ_NR_ is 100 times shorter below 50 K compared to that in S2, indicating the nonradiative recombination rate is more significant in S1 compared to S2. This finding further supports our hypothesis that the droop mechanism is primarily due to the overflow effect, further confirming our findings from PD-TRPL, TDPL, and the IQE droop analyses.

The findings reported here indicate that the inclusion of two strain-engineering regions (L1 and L2) further enhances the optical efficiency of the LED with SLs in S2 structure (rather than a single SL) in L2 in S1 structure for several reasons. Figure 5g shows the possible mechanism that enhances the IQE and its droop of S2 compared to that in S1. At high G, carrier transfers from the main MQW state to populate the higher-energy states in the sub-QWs at the V-pit sidewalls followed by return back to repopulate of the main MQW states (as illustrated in the schematic of Figure 5g) via the MQWs located near the V-pits that are characterized by bottom-to-top gradual increase of In contents, resulting in further blueshift and greater fwhm as *G* increases in S2 compared to S1, as shown. This mechanism is supported by an enhanced IQE as well as droop behavior in S2.

First, the location at which V-pits originate as well as the thickness of their sidewalls has a marked impact on their size. For instance, V-pits that are initiated below SLs are larger than those that emerge near the surface,^22^ as the formation of larger V-pits below the SLs region is assisted by L1. The deeper and larger-sized V-pits prevent carriers from moving laterally into TDs as their sidewalls form high-potential barriers that do not permit carriers to recombine nonradiatively via the defect states.^48^ Thus, much larger V-pit sidewalls (comprising MQWs + 12 SLs) due to SL inclusion are obtained in S2 compared to that in S1 (MQWs + 1RL), leading to lower nonradiative recombination contribution in S2. Second, incorporation of SLs into S2 facilitates more homogeneous In inclusion into MQWs compared to S1, where In-rich segregations increase the number of nonradiative recombination centers, thereby reducing the IQE value and affecting the droop mechanisms. Third, the gradual In content increase in the [0001] direction. MQWs (from top to bottom) near V-pits facilitate repopulations between the MQWs and the subwells related to ultrathin V-pit sidewalls via an overflow process, as shown in Figure 5g, which can lead to the droop shape as well as contribute to greater IQE efficiency in S2.

Finally, deeper V-pits enhance the quality of the underlying *n*-GaN layer as the V-pit density increases within it, which in turn helps relieve strain and reduces TD density, resulting in a smaller number of nonradiative centers.^18,49^ These complex processes ensure that more carriers recombine radiatively within active regions, leading to a more efficient light emission and thus higher IQE for S2.

### 3.5 Electroluminescence (EL) Measurements

To elucidate our findings and conclusion, we carried out RTEL measurements for both LEDs (S1 and S2) that were carried out under the same conditions to compare their performance, and the findings are presented in Figure 6a–c. As shown in Figure 6a, both samples exhibit a blue EL peak, located at ∼475 and 471 nm, for LED S1 and S2, respectively. However, S2 shows a notably higher EL intensity compared (∼1.6 times) to S1, further confirming the more dominant radiative recombination of the MQW emission produced by S2. It is also evident from Figure 6b that, in S2, the total light optical output power as a function of the electrically pumped injected carriers is considerably enhanced compared to that of S1. For example, at an injection current of ∼100 mA, S2 yields an output power of ∼125 mW, which is ∼66% greater than 75 mW produced by S1.

**Figure 6:**
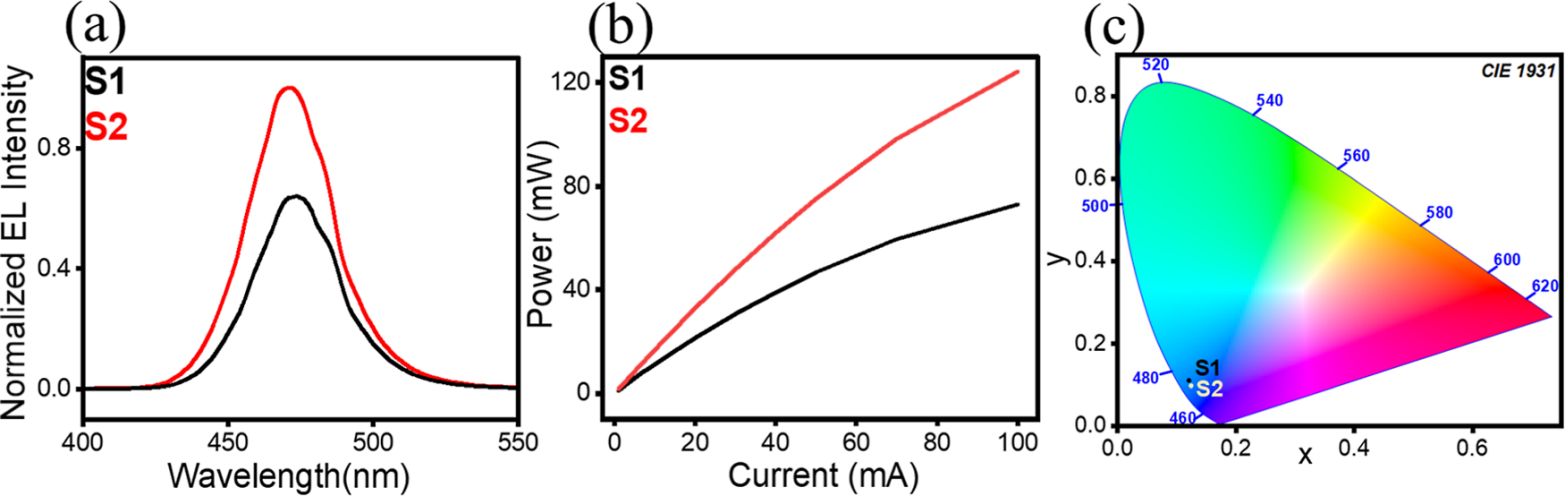
(a) RT EL spectra for both LEDs; (b) optical power versus injection current from (0 to 100 mA); and (c) color coordinates in the CIE 1931 diagram for both LEDs.

To further confirm the observed efficiency improvement, External Quantum Efficiency (EQE) was calculated using the eq 4 below^19^

5 EQE=PhvIe=pλI×ehc=pλ1240×I

where *P* is the optical power, λ is the emission wavelength, and *I* is the injection current. According to eq 4, the EQE of S2 is 63%, showing a substantial optical enhancement compared to that of S1 (43%). Consequently, in the CIE 1931 diagram shown in Figure 6c, since S2 has a noticeably lower y value than S1, it plots closer to the spectral location in the violet–blue direction, which corresponds to a more saturated (purer) blue; especially, the width of the EL peak of S2 is narrower (Δfwhm = ∼4 nm) than that of S1. These combined results indicate that the structural or material optimizations in S2 effectively suppressed nonradiative recombination and improved carrier confinement within the active layers of LED S2, concurring with our IQE findings.

## 4 Conclusions

We examined the combined effects of pre-multilayers in L1 and SLs in L2 on significantly improving the carrier dynamics and optical properties of S2 compared to S1 LED structures characterized by a single SL in L2. The L1 layers promoted the formation of deep V pits between L1 and L2. This resulted in the development of large, deep V-pits and higher-quality GaN below the active layer. For LED structures with 12 SL pairs, we observed a gradual decrease in the In concentration in the MQWs located near the V-pits as they moved upward from the bottom layer. This decrease continued until saturation was reached, which occurred only in the regions around the V pits. This gradual reduction in In concentration was not seen in areas away from the V-pits or in the S1 structure (which had a single SL). The gradual decrease in the In concentration can help facilitate sufficient overflow processes from the main MQWs to the sub-QWs on the V-pit sidewalls. This overflow process along with the band-filling process assists in repopulating the higher energy states of the carriers between the MQWs and the sub-QWs, resulting in superior IQE and EQE values for S2 LED compared to that of S1 LED. This work highlights how varying strain-engineering regions can improve the internal IQE and EQE, which can ultimately enhance the performance of other InGaN-based light-emitting devices.

## Supplementary Material



## Notes

Experimental setups of SIMS; asymmetric RSM scans; contributions of radiative and nonradiative for fast decay; and PL spectra for both structures using energy above the bandgap (PDF)
