# Supplementary material for: Enhanced Optical Efficiency and Carrier Dynamics in InGaN/GaN Light-Emitting-Diode Structures through Combination of Superlattice- and Prewell-Strain Control Strategy
Source: ACS Appl Mater Interfaces. 2026 Jun 25;18(26):36989–99. doi: 10.1021/acsami.6c06408 (PMC13352510; doi:10.1021/acsami.6c06408)
Supplement: Supplementary file 1 [file am6c06408_si_001.pdf]

Supporting Information

Enhanced optical efficiency and carrier dynamics in InGaN/GaN light-emitting-diode structures through combination of superlattice- and pre-well-strain control strategy

Fatimah Alreshidi[1], Lih-Ren Chen[2], Hadeel Alamoudi[1], Dhaifallah R. Almalawi[3], Nimer Wehbe[4], Georgian Melinte[4], Tien-Chang Lu[2], Iman S. Roqan[1, *]

[1]*Physical Sciences and Engineering Division, King Abdullah University of Science and Technology (KAUST), Thuwal 23955-6900, Saudi Arabia.*

[2]*Department of Photonics, College of Electrical and Computer Engineering, National Yang Ming Chiao Tung University, Hsinchu 30010, Taiwan*

[3]*Department of Physics, College of Science, Taif University, Taif 21944, Saudi Arabia*

[4]*Imaging and Characterization Core Laboratory, King Abdullah University of Science and Technology, Thuwal 23955-6900, Saudi Arabia*

Corresponding author: iman.roqan@kaust.edu.sa

S1: Secondary Ion Mass Spectrometry (SIMS)

Experimental setup:

Depth profiling experiments were performed on a Dynamic SIMS instrument from Hiden analytical company (Warrington-UK) operated under ultra-high vacuum conditions, typically 10^{-9} mbar. A continuous Ar^+ beam was employed at 2.5 keV to sputter the surface while the selected ions ascribed to Ga, N and In were sequentially collected using a MAXIM spectrometer equipped with a quadrupole analyzer. Prior to data acquisition, key experimental parameters—primarily the primary ion energy—were optimized. The sputtered area had an estimated raster size of 750 × 750 μm^2. To minimize edge effects during sputtering, SIMS signals were collected from a smaller central region of approximately 75 × 75 μm^2, defined using electronic gating. Sputtering time was converted to depth by measuring the resulting crater depth at the end of the profiling process, using a DektakXT stylus profiler (Veeco).

SIMS data:

To assess the in-depth quality of the sample—particularly the multi-quantum well (MQW) structure—depth profiling was performed using SIMS. The ion signals corresponding to In and Ga were recorded and are presented as a function of depth in Figure (1). At the onset of sputtering, a notable decay of In and Ga signal intensity is observed within the first 15 – 20 nm, attributed to pronounced surface oxidation. Despite this phenomenon, we can distinguish three series of InGaN MQW structures clearly visible through the oscillatory pattern of the indium signal. The MQW group detected between the surface down to approximately 85 nm depth corresponds to nine distinct MQW layers for both LED structures. The initial group is immediately followed by a single InGaN/GaN SL for (S1), and twelve pairs of InGaN/GaN SLs (S2) in L2 region, which is between 85 and 165 nm depth. These subsequent layers exhibit reduced inter-layer spacing (indicating thinner InGaN layers) and lower indium signal intensity,

suggesting a probable decrease in indium content compared to the MQW series. Additionally, the slight decrease in the indium signal with depth observed in both series is likely an artifact caused by sputtering-induced damage, which leads to increased surface roughness as depth increases.[1] Consequently, signals from the thin MQW layers become more difficult to resolve under these conditions. Following the second MQW series, the indium signal drops by more than an order of magnitude before rising again in the final MQW region, detected between 250 nm and 365 nm. For both LED structures, this final series consists of five pre- strain engineering multilayers (L1) exhibiting the lowest indium signal intensity, but the largest inter-layer spacing compared to the first two series. Overall, the SIMS data confirms the successful fabrication of the MQW structure.

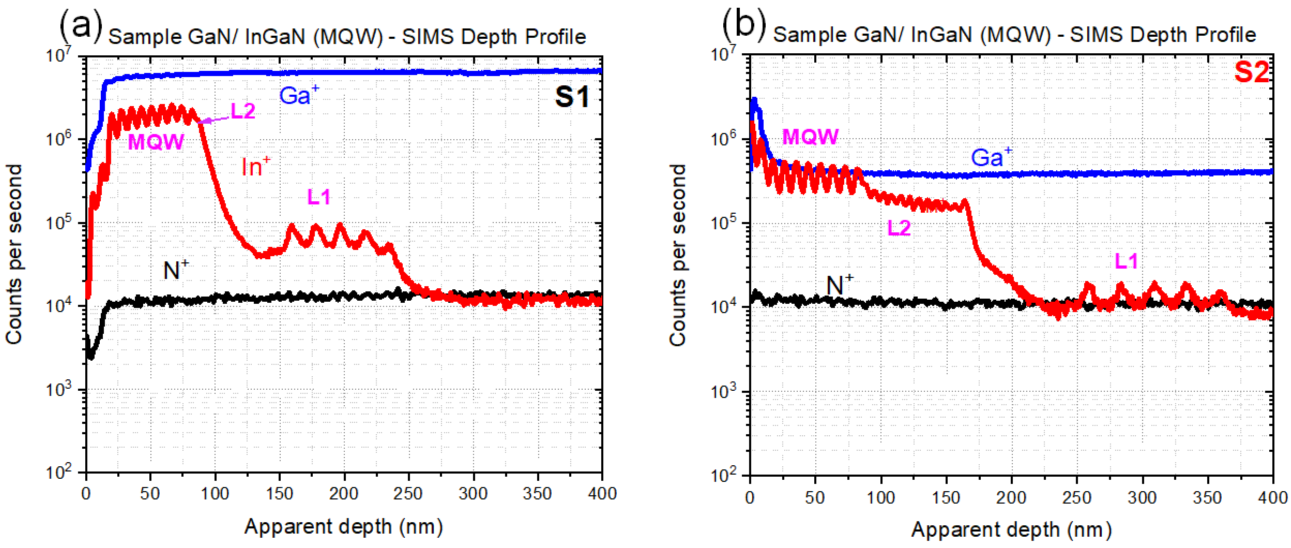

Figure S1: SIMS signals of Ga^+ (blue), In^+ (red) and N^+ (black) ions as functions of depth obtained for (a) S1 and (b)S2 structures.

S2: Raman spectra

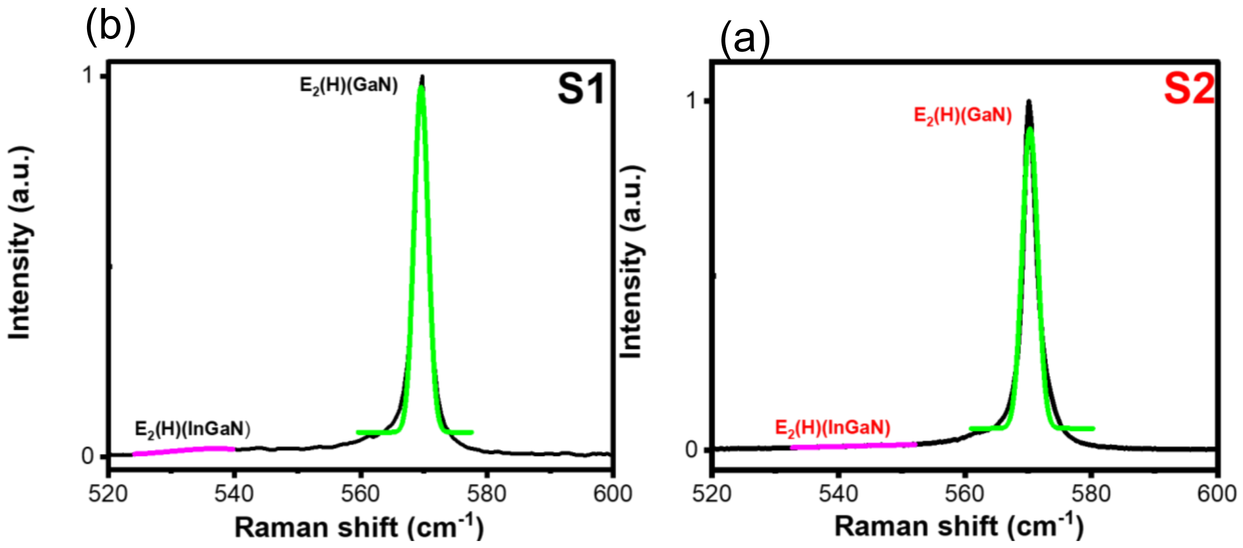

Figure S2: RT μ-Raman spectra for (a) S1 and (b) S2 structures.

S3: Reciprocal Space Mapping (RSM)

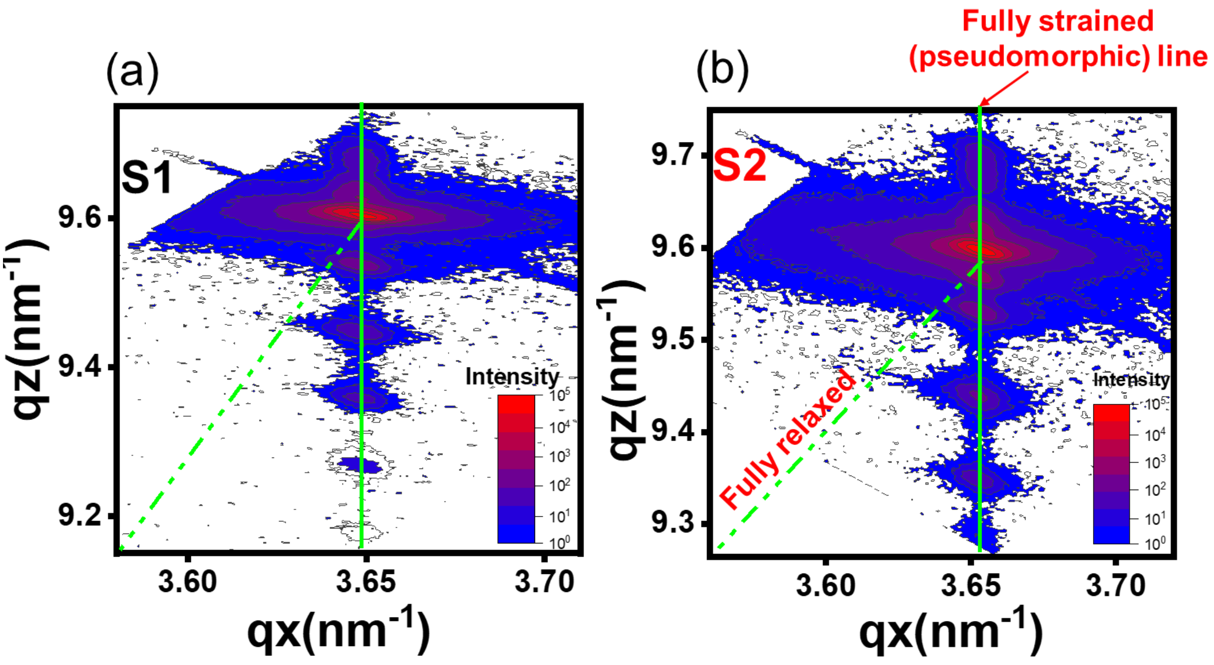

Figure S3. Asymmetric RSM scans along the (105) reflection for (a) S1, (b) S2 (green solid indicates line Fully pseudomorphic, and the green dashed line indicates fully relaxed).

S4: Power dependent-PL at RT

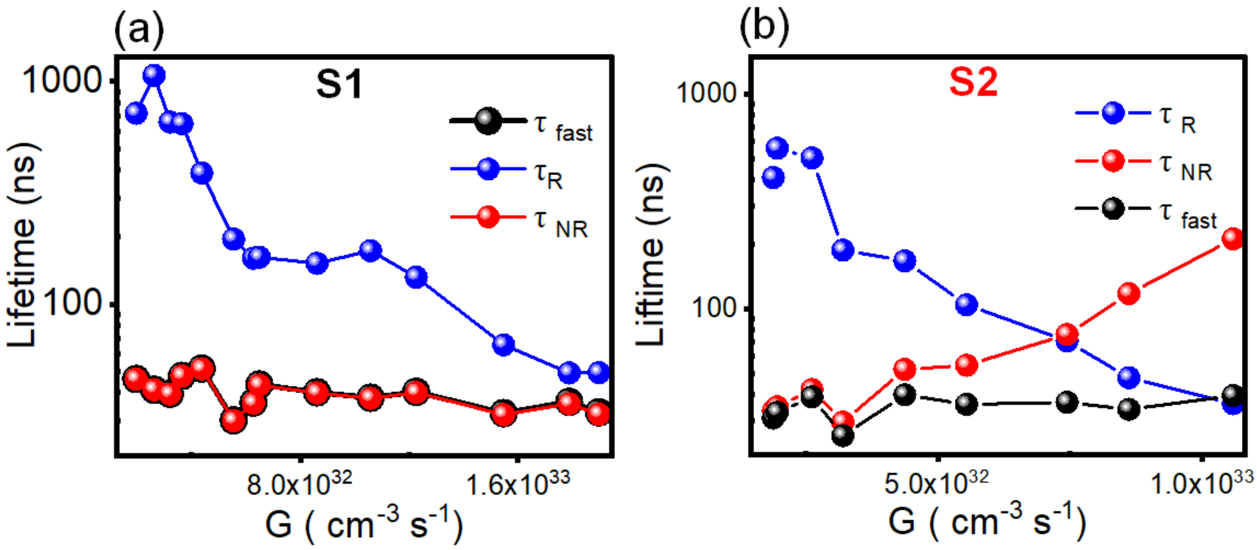

Figure S4: Total, Radiative and non-radiative recombination lifetimes as functions of carrier generation (G) for (a) S1 and (b) S2 structures.

S5: Temperature dependent-PL

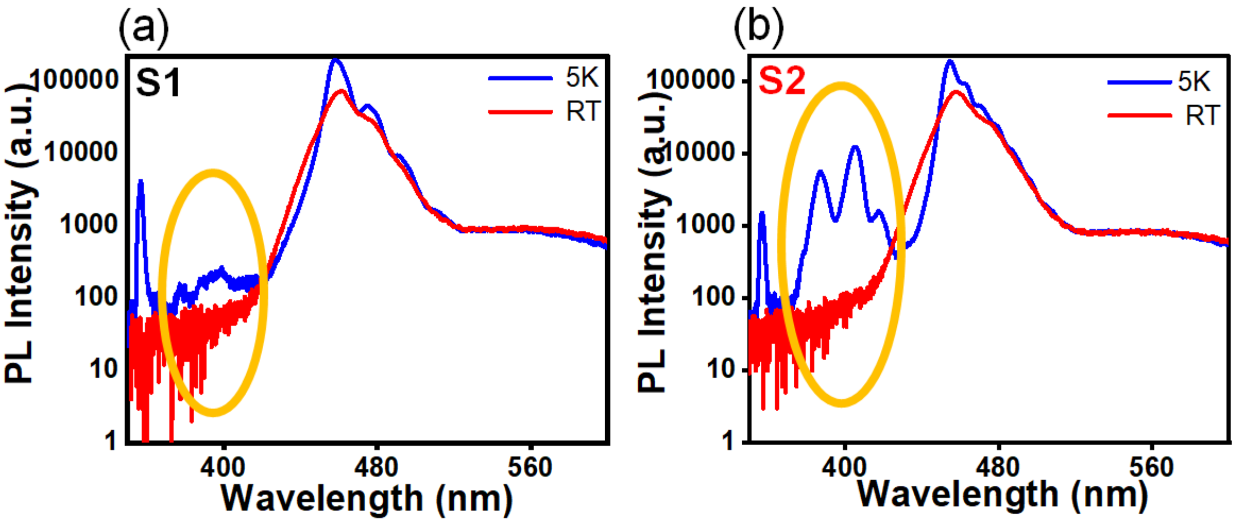

Figure S5: RT and 5K PL spectra of InGaN/GaN MQWs excited by 325 nm for (a) S1 and (b) S2 structures. (The yellow circled areas showing the V-pits related emission).

Reference:

1- SIMS: A Practical Handbook for Depth Profiling and Bulk Impurity Analysis, R.G Wilson, F.A.Stevie and C.W.Magee, John Wiley and Sons NY 1989.
